# The rate of patients screened positive for gaming disorder/Internet gaming disorder among adolescents with mental health issues assessed by two screening tests: A nine‐item screening test for GD (GAMES Test) and the Ten‐Item Internet Gaming Disorder Test (IGDT‐10)

**DOI:** 10.1002/pcn5.70080

**Published:** 2025-03-13

**Authors:** Masaru Tateno, Takanobu Matsuzaki, Ayumi Takano, Yukie Tateno, Takahiro A. Kato, Susumu Higuchi

**Affiliations:** ^1^ Tokiwa Child Development Center, Tokiwa Hospital Sapporo Japan; ^2^ Department of Neuropsychiatry School of Medicine, Sapporo Medical University Sapporo Japan; ^3^ Department of Psychiatry National Hospital Organization Kurihama Medical and Addiction Center Yokosuka Japan; ^4^ Department of Drug Dependence Research National Center of Neurology and Psychiatry Kodaira Japan; ^5^ Department of Neuropsychiatry Graduate School of Medical Sciences, Kyushu University Fukuoka‐city Japan

**Keywords:** attention deficit hyperactivity disorder (ADHD), gaming disorder, Internet gaming disorder, neurodevelopmental disorders, screening test

## Abstract

**Aim:**

The aim of this study is to estimate the rate of patients who screened positive for gaming disorder (GD) in ICD‐11 among adolescents with psychiatric and/or developmental disorders by using two screening tests: a nine‐item screening test for GD, GAMing Engagement Screening test (GAMES Test), and the Ten‐Item Internet Gaming Disorder Test (IGDT‐10).

**Methods:**

Subjects were 257 adolescent patients attending a psychiatric clinic in Sapporo, Japan. They were asked to complete two questionnaires. The total score on the IGDT‐10 was calculated by two different scoring methods, original version (OV) and modified version (MV). The three groups were also compared on the basis of their clinical diagnoses.

**Results:**

Of the 203 respondents, 42 (20.7%) screened positive using the GAMES Test. With respect to the IGDT‐10, only eight (3.9%) screened positive using the IGDT‐10 OV scoring, while 55 (27.1%) screened positive using the IGDT‐10 MV. The most notable finding was that the mean total scores on the GAMES Test and the IGDT‐10 MV were significantly higher in the attention deficit hyperactivity disorder group than in the other two groups (depression and autism spectrum disorder).

**Conclusion:**

The results of this study showed that adolescents with mental health problems had a higher rate of screening positivity on self‐rated screening tools for GD than the general population. Because excessive gaming has a wide range of adverse effects on adolescents' mental health, early detection of probable GD is crucial. Screening for GD should be part of routine clinical practice.

## INTRODUCTION

The Internet has become a social infrastructure and an indispensable part of our daily lives. Lifestyles and the way society is organized have been transformed by the spread of the Internet. According to a large‐scale survey conducted by the Japanese government, the Internet usage rate is almost 100% for age groups ranging from young teens to those under 60 years.^1^ The Internet has become an essential tool in many areas of our lives.

One recent change in Internet use is the decrease in the age at which people begin using the Internet. The results of a large‐scale survey on children's Internet use revealed that 30% of children aged 1 year, 60% aged 2 years, 80% aged 5 years, and 90% aged 7 years use the Internet daily.^2^ Internet use from an early age, such as in infancy, is a problem that requires attention. A cohort study analyzed data from mother–child dyads in a large birth cohort in Japan (*n* = 84,030) and investigated the effects of screen time, including smartphones and tablets, during infancy.^3^ The study revealed that the prevalence of autism spectrum disorder (ASD) at 3 years of age was 392 per 100,000 (0.4%). In addition, logistic regression analysis demonstrated that among boys only, when no screen time was the reference, the adjusted odds ratios for an ASD diagnosis were 1.38 for boys with less than 1 h of screen time, 2.16 for 1 to less than 2 h, 3.48 for 2 to less than 4 h, and 3.02 for more than 4 h, respectively. These findings suggest that excessive Internet use from an early developmental stage may have serious negative effects on children's social and language development. Furthermore, studies have shown that early gaming habits are risk factors for pathological gaming.^4^

Gaming is one of the most popular leisure activities among children and adolescents in Japan. As Internet access has advanced, it has become commonplace for young teenagers to play online games. The Cabinet Office of Japan has conducted an annual survey titled “Survey on the Internet Use Environment of Youth” since 2009, when problems related to the Internet began to be pointed out. According to the survey results, the percentage of children and adolescents using the Internet for games was 74.9% in 2017, and has steadily increased every year since, reaching 79.9% in 2020 and 85.5% in 2023.^5^ By playing online gaming, children can collaborate with their friends in an online virtual space and enjoy chatting while playing games. Consequently, the time spent on gaming increased. In clinical practice, the number of children and adolescents presenting to psychiatric clinics with gaming‐related problems is increasing. In a survey of child and adolescent psychiatrists (*n* = 159), 88.1% of respondents agreed that the prevalence of excessive gaming and Internet‐related problems among children is likely to continue rising.^6^

In 2013, preliminary diagnostic criteria for Internet gaming disorder (IGD) were proposed in Section III, Conditions for Further Study in the Fifth Edition of the *Diagnostic and Statistical Manual of Mental Disorders* (DSM‐5) by the American Psychiatric Association.^7^ The diagnostic criteria for IGD consisted of nine criteria: (1) preoccupation with gaming; (2) withdrawal symptoms; (3) increased tolerance to gaming; (4) unsuccessful attempts to control participation in gaming; (5) loss of interest in other hobbies or activities; (6) excessive gaming despite negative consequences; (7) deception about gaming activities towards others; (8) use of gaming as an escape or relief from a negative mood; and (9) jeopardized or lost relationships, jobs, or educational or career opportunities. To diagnose IGD, five or more of the nine items must be met. The diagnosis of IGD is limited to Internet games, and gambling should be excluded. The proposed diagnostic criteria for IGD have led to a rapid increase in the research on excessive pathological gaming.

In 2019, the World Health Organization (WHO) adopted the 11th revision of the *International Classification of Diseases* (ICD‐11), identifying excessive gaming as a psychiatric disorder.^8^ Gaming disorder (GD) is characterized by a pattern of persistent or recurrent gaming behavior, manifested by (1) impaired control over gaming, (2) increasing priority given to gaming to the extent that gaming takes precedence over other life interests and daily activities, and (3) the continuation or escalation of gaming despite the occurrence of negative consequences. This pattern of gaming behavior results in marked distress or significant impairment in important areas of functioning. Gaming behavior and other features are normally evident over a period of at least 12 months for a diagnosis to be assigned, although the required duration may be shortened if all diagnostic requirements are fulfilled and symptoms are severe. It should be noted that the diagnostic guidelines for GD state that it is applicable to either online or offline gaming, that is, gaming without Internet access.

Excessive gaming can lead to mental health problems. In addition, psychiatric and neurodevelopmental disorders are known risk factors of GD.^9^, ^10^, ^11^ In other words, comorbid psychiatric disorders can be both the cause and the result of GD. Accumulating evidence has revealed that excessive gaming is closely related to neurodevelopmental disorders, such as attention deficit hyperactivity disorder (ADHD) and autism spectrum disorder (ASD).^6^, ^9^, ^12^, ^13^

Our previous surveys suggested that children and adolescents with mental health issues have a higher risk of developing GD.^6^, ^14^ Early detection and intervention of probable GD are important for the prevention of severe GD. Accordingly, several screening tests and assessment tools have become available.^15^ Of these, two screening tests used in clinical settings in Japan are the Ten‐Item Internet Gaming Disorder Test (IGDT‐10)^16^ and the nine‐item short screening test for ICD‐11 gaming disorder, GAMing Engagement Screening test (GAMES Test).^17^ The Japanese versions of these two self‐administered screening tests are available on the National Hospital Organization Kurihama Medical and Addiction Center website (https://kurihama.hosp.go.jp/).

This study aimed to estimate the rate of patients who screened positive for GD/IGD and compare the differences in the positive rates between these tests among adolescents with psychiatric and/or developmental disorders. In addition, we compared the differences in the severity of addiction to gaming among three diagnostic groups reported to be frequently comorbid with GD in previous studies: depression, ASD, and ADHD.

## METHODS

### Study design

This was a single‐site cross‐sectional study.

### Study participants

The study participants were recruited from the outpatient clinic of the Tokiwa Child Development Center in Sapporo, Japan. Previous studies have reported that depression and neurodevelopmental disorders are frequently comorbid with GD.^6^, ^9^, ^10^ Given that these studies have identified depression and neurodevelopmental disorders as the most common comorbidities of GD, and that a sufficient number of patients is necessary for comparison by diagnostic group, we invited only patients whose primary diagnosis was depression, ASD, or ADHD to participate in this study. All eligible adolescents, ranging from 10 to 18 years of age, were invited to participate in the study, except for patients with a co‐morbidity of intellectual disability of moderate or greater severity, as this study was based on a self‐administered questionnaire. In other words, subjects were selected through a continuous sampling method. Two child and adolescent psychiatrists (CAPs) (M.T. and Y.T.) obtained consent from the examination room and handed the participants the questionnaire. Two experienced CAPs made clinical diagnoses of the subjects according to the diagnostic criteria of the DSM‐5. The participants were asked to complete the questionnaire. The time required to complete the questionnaire was typically between 5 and 10 min.

### Measures

#### Study questionnaire

The study questionnaire consisted of questions about demographics (including age and sex), gaming behavior (length of gaming on weekdays and weekends), presence or absence of school refusal, and two screening tools for GD in ICD‐11 and IGD in DSM‐5, as described below. In Japan, school refusal is defined as being absent from school for 30 or more days in a year.^18^

#### The Ten‐Item Internet Gaming Disorder Test

The IGDT‐10, a self‐rating scale, was developed by Király et al.^16^ The study subjects for the development of IGDT‐10 were 4887 Hungarian online gamers (aged 14–64 years, mean age 22.2 ± 6.4 years, 92.5% male) recruited using social media and gaming‐related websites. IGDT‐10 was designed to screen for IGD according to the diagnostic criteria described in Section III of the DSM‐5.^8^ The IGDT‐10 consists of 10 questions regarding the frequency of gaming‐related problems during the previous 12 months on a three‐point Likert scale: 0 = *Never*, 1 = *Sometimes*, and 2 = *Often*. The IGDT‐10 assesses functional impairment via two questions, Q‐9 (risked or lost a significant relationship because of gaming) and Q‐10 (jeopardized your school or work performance because of gaming), that belong to the same criterion of IGD of DSM‐5, item‐9 (jeopardized or loss of a significant relationship, job or educational or career opportunity because of participation in Internet games). Therefore, selecting *Often* on either Q‐9 or Q‐10, or on both items, contributes only 1 point to the score. Thus, the total IGDT‐10 score ranged from 0 to 9. If the total score was five or higher, the respondents were diagnosed with IGD.

#### Two scoring methods of IGDT‐10

In this study, we compared the total scores of the original (OV) and revised (RV) versions of the IGDT‐10.

The original cutoff points proposed by the developer of the IGDT‐10 were five or more responses of 2 = *Often* (original version).^16^ That is, it is considered positive only when *Often* is selected. As mentioned earlier, although the IGDT‐10 consists of 10 questions, the maximum score is 9 points because it assesses dysfunction in daily life using two questions, Q9 and Q10. In this study, the original scoring method was defined as IGDT‐10 OV.

In 2022, Mihara et al. proposed a less stringent scoring method: responses of either *Sometimes* or *Often* were regarded as affirmative, and a total score of 5 or more was treated as screen‐positive on the IGDT‐10.^19^ The total number of respondents in the initial survey conducted in Japan was 5096, aged 10–29 years old. After performing several screenings, face‐to‐face interviews were conducted with 325 participants, and a clinical diagnosis of IGD was made by medical professionals using the Japanese version of the Structured Clinical Interview for IGD, which was published as supplementary material to the paper.^19^ The results of Mihara's study demonstrated that the sensitivity and specificity were sufficiently high, and receiver–operating characteristic analysis revealed a higher screening performance of the proposed modified scoring method than that of the original scoring method. The proposed cutoff for the modified scoring to screen for GD was 5 points, which is the same as the original scoring. In this study, we referred to this modified scoring method as the IGDT‐10 MV.

#### GAMing Engagement Screening test (GAMES Test)

A nine‐item short screening test for ICD‐11 gaming disorder, GAMing Engagement Screening test (GAMES Test), was developed as a screening test for GD in ICD‐11 by Higuchi et al.^17^ The GAMES Test included nine items in total, including eight items representing four criteria of GD followed by one question about the duration of gaming. The four GD criteria were (1) impaired control over gaming, (2) increasing priority given to gaming, (3) continuation or escalation of gaming despite adverse consequences, and (4) significant impairment in functioning. Respondents were asked to answer two questions for each of the four GD criteria. There were two ways to answer the questions in the GAMES Test: *Yes* or *No* for the first eight questions and by choosing 0 (less than 2 h), 1 (at least 2 h but less than 6 h), or 2 (6 h or longer) for the question asking about the time spent gaming each weekday. The total score of the GAMES Test ranged from 0 to 10, and the proposed cutoff point was 5 or more based on the results of their large‐scale survey, including face‐to‐face interviews using a semi‐structured interview form, as described in the IGDT‐10 MV method.

### Statistical analysis

All statistical analyses were performed using SPSS Version 25.0 (IBM Corp., 2017). The *t*‐test, residual analysis in the chi‐square test and analysis of variance (ANOVA) post‐hoc Tukey–Kramer test were used based on the characteristics of the data. Statistical significance was defined as *p* < 0.05.

### Ethics

This study was approved by the ethics committee of the Tokiwa Hospital (TH‐201026). Informed consent was obtained from all the participants and their guardians. The study procedures were carried out in accordance with the Declaration of Helsinki.

## RESULTS

### Screen‐positive rate and the mean total score of the GAMES Test, IGDT‐10 OV, and IGDT‐10 MV

The results are presented in Table 1. Forty‐one non‐game players and 13 respondents with missing answers were excluded from the 257 respondents. Among 203 respondents, 42 (20.7%) screened positive using the GAMES Test. Regarding IGDT‐10, when we applied the original version provided by the developer of the IGDT‐10, only eight (3.9%) respondents screened positive. On the other hand, according to the modified version, which regards 1 = *Sometimes* as affirmative, 55 (27.1%) screened positive. The difference in the positivity rates between the two scoring methods was approximately seven times.

**Table 1:** Characteristics of the subjects and the summary of the results.

		Overall (*n* = 203)	Male (*n* = 141)	Female (*n* = 62)	*p* value
Age (years)		13.6 ± 2.6	13.5 ± 2.5	13.9 ± 2.8	0.4340
Time of gaming	Weekdays	2.53 ± 1.94	2.66 ± 2.03	2.24 ± 1.68	0.1311
	Weekends	4.23 ± 3.16	4.49 ± 3.38*	3.65 ± 2.50	0.0493*
School refusal (%)		44 (21.7)	29 (20.6)	15 (24.2)	0.5636
GAMES Test	Total score	2.6 ± 2.2	2.6 ± 2.2	2.6 ± 2.3	0.8602
	positive (%)	42 (20.7)	27 (19.1)	15 (24.2)	0.4138
IGDT‐10 MV	Total score	3.4 ± 2.3	3.5 ± 2.1	3.1 ± 2.6	0.2723
	positive (%)	55 (27.1)	41 (29.1)	14 (22.6)	0.3374
IGDT‐10 OV	Total score	1.0 ± 1.5	1.0 ± 1.4	0.9 ± 1.6	0.7733
	positive (%)	8 (3.9)	3 (2.1)	5 (8.1)*	0.0452*

*Note*: A *t‐*test was applied to the two‐group comparison of the mean age between males and females. The chi‐square test was used to compare the other groups. Asterisks indicate statistical significance (*p < 0.05). Age is expressed as the mean ± standard deviation. The figures in parentheses represent the percentage of the total.

Abbreviations: GAMES Test, a nine‐item short screening test for ICD‐11 gaming disorder; IGDT‐10, Ten‐Item Internet Gaming Disorder Test; MV, modified version; OV, original version.

Regarding the consistency of the results, 73.8% (31 out of 42) of the GAMES‐Test‐positive respondents were screened as positive by the IGDT‐10 MV, whereas only 16.7% (seven out of 42) were positive based on the IGDT‐10 OV.

Statistical analyses (chi‐square test) revealed sex differences only in the positive IGDT‐10 OV group. Although the maximum total score for the IGDT‐10 OV was 9, the highest score for the respondents in this study was 7.

In terms of gaming duration, males played longer than females on weekdays and holidays. However, a statistically significant difference was found only on the weekends (*t*‐test).

School refusal is a common problem among adolescents with mental health issues, at an overall rate of approximately 20%. The rate was higher among females (24.2%) than males (20.6%). The mean scores of the two screening tests with two different scoring methods for IGDT‐10 were compared between the two groups (presence and absence of school refusal), but no significant differences were found: GAMES Test, 2.75 vs 2.54, *p* = 0.5911; IGDT‐10 MV, 3.48 vs 3.33, *p* = 0.6846; IGDT‐10 OV, 1.16 vs 0.89, *p* = 0.3281.

### The correlation between the total score of the GAMES Test and IGDT‐10 MV/IGDT‐10 OV

**Figure 1:**
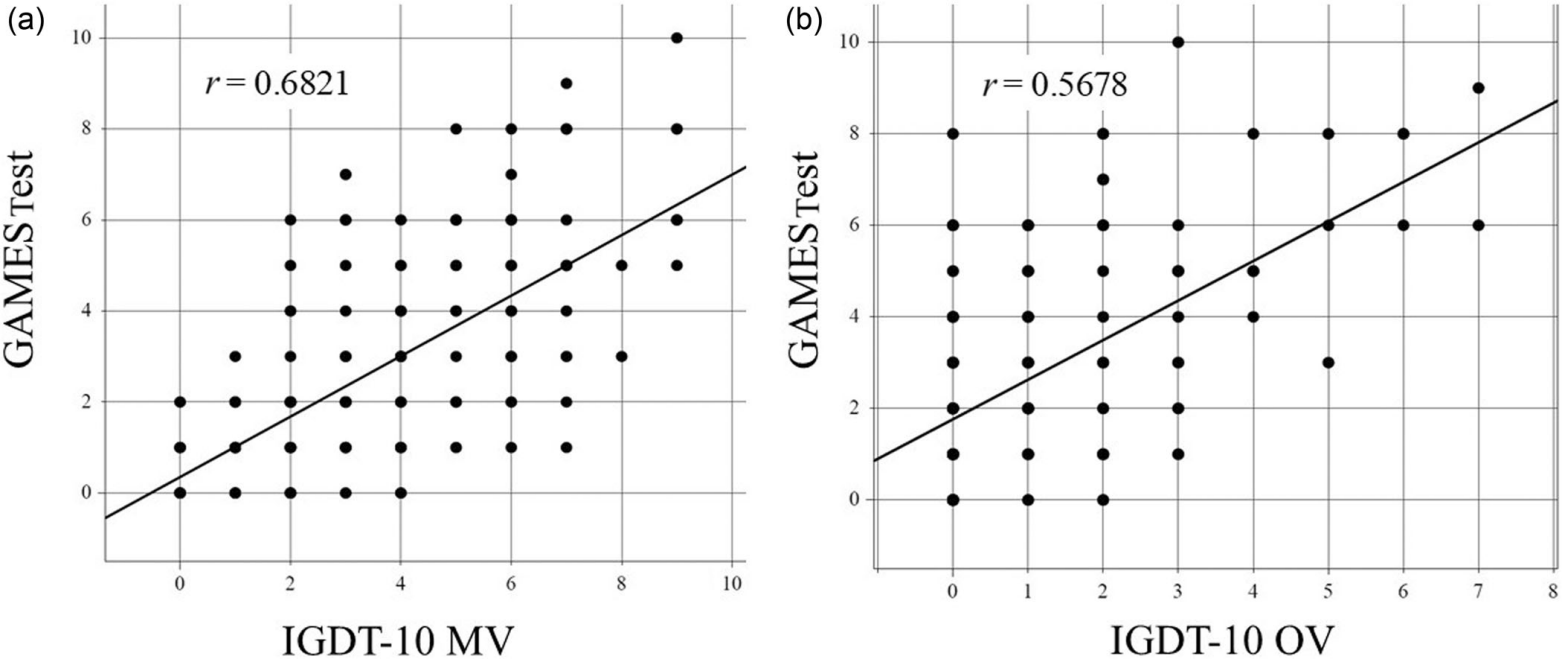
The correlation between the total score of a nine‐item short screening test for ICD‐11 gaming disorder (GAMES Test) and the (a) Ten‐Item Internet Gaming Disorder Test (IGDT‐10) modified version (MV) and (b) IGDT‐10 original version (OV). The GAMES Test and IGDT‐10 MV exhibited stronger correlations than the GAMES Test and IGDT‐10 OV. *r* is the Pearson correlation coefficient.

We investigated the correlation between the total GAMES Test score and IGDT‐10 MV/IGDT‐10 OV. The results are shown in Figure 1. Although the IGDT‐10 is based on the diagnostic criteria of IGD in the DSM‐5 and the GAMES Test is based on the diagnostic guidelines of GD in the ICD‐11, a strong correlation was found between the IGDT‐10 and the GAMES Test. The Pearson correlation coefficient (*r*) was 0.6821 (95% confidence interval [CI]: 0.6009–0.7494) for the IGDT‐10 MV and GAMES Test and 0.5678 (0.4666–0.6544) for the IGDT‐10 OV and GAMES Test. Consequently, the correlation between the GAMES Test and IGDT‐10 MV was stronger than that between the GAMES Test and IGDT‐10 OV.

### The three‐group comparison based on clinical diagnosis: Depression, ASD, and ADHD

The subjects were then divided into three groups based on their clinical diagnosis confirmed by CAPs through regular and continuous follow‐up for longer than 3 months.

Among the three diagnostic groups, we compared their daily gaming time on weekdays and weekends, the presence and absence of school refusal, and the results of the two questionnaires with modified scoring methods for the IGDT‐10. The results are presented in Table 2. The mean age of the depression group was significantly higher than that of the other two groups. Statistical analyses revealed no significant correlation between age and the GAMES Test (*r* = −0.0422, *p* = 0.5504) or IGDT‐10 OV (*r* = 0.0822, *p* = 0.2438). However, a statistically significant correlation was identified between age and total score on the IGDT‐10 MV (*r* = −0.1432, *p* = 0.0416), although the correlation was relatively weak. Higher age in the depression group was deemed negligible when taken together. There were no significant differences in the daily gaming times on weekdays and weekends or in the positive rate of each screening tool among the three groups.

**Table 2:** Comparison of the results of the three diagnostic groups.

		Overall (*n* = 203)	Depression (*n* = 39)	ASD (*n* = 98)	ADHD (*n* = 66)	*p* value
Age (years)		13.6 ± 2.6	14.6 ± 2.0*	13.6 ± 2.5	12.9 ± 2.4	0.0017*
Time of gaming	Weekdays	2.53 ± 1.94	2.21 ± 1.62	2.56 ± 2.15	2.67 ± 1.77	0.4768
	Weekends	4.23 ± 3.16	3.58 ± 2.14	4.05 ± 2.77	4.89 ± 4.02	0.0850
School refusal (%)		44 (21.7)	18 (45.0)*	20 (20.4)	6 (9.1)	0.0001*
GAMES Test	Total score	2.6 ± 2.2	2.4 ± 2.4	2.3 ± 1.9	3.2 ± 2.5*	0.0231*
	positive (%)	42 (20.7)	7 (17.9)	15 (15.3)	20 (30.3)	0.0770
IGDT‐10 MV	Total score	3.4 ± 2.3	2.9 ± 2.3	3.2 ± 2.1	3.9 ± 2.4*	0.0376*
	positive (%)	55 (27.1)	9 (23.1)	25 (25.5)	21 (31.8)	0.5519
IGDT‐10 OV	Total Score	1.0 ± 1.5	0.8 ± 1.8	0.9 ± 1.3	1.2 ± 1.5	0.3920
	positive (%)	8 (3.9)	4 (10.3)	1 (1.0)*	3 (4.5)	0.0412*

*Note*: A *t*‐test was applied to the two‐group comparison of the mean age between males and females. The chi‐square test was used for comparisons with other groups. Asterisks indicate statistical significance (*p < 0.05).

Age is expressed as mean ± standard deviation. Figures in parentheses represent percentages of the total.

Abbreviations: ADHD, attention‐deficit hyperactivity disorder; ASD, autism spectrum disorder; GAMES Test, a nine‐item short screening test for ICD‐11 gaming disorder; IGDT‐10, Ten‐Item Internet Gaming Disorder Test; MV, modified version; OV, original version.

## DISCUSSION

The fact that GD is considered a psychiatric disorder in the ICD‐11 is of great interest to society worldwide. Given the conceptualization of GD as a psychiatric disorder, many parents are concerned about their children's excessive gaming. GD is listed in the category of “Disorders due to addictive behaviors” along with gambling disorder under the higher category entitled “Disorders due to substance use or addictive behaviors” in the ICD‐11.^8^ Unlike other addictive disorders, many patients with GD are children and adolescents.

The results of this study showed that adolescents with mental health problems had a higher rate of screening positivity on self‐rated screening tools for GD than the general population. The percentage of patients who tested positive for the GAMES Test, IGDT‐10 OV, and IGDT‐10 MV were 20.7%, 3.9%, and 27.1%, respectively. A national survey of the general population in Japan aged in their teens and twenties reported that 5.1% screened positive in the GAMES Test.^17^ Similarly, a previous national study revealed that 1.8% and 11.3% of Japanese teenagers and people in their twenties (*n* = 5096) screened positive for the IGDT‐10 OV and IGDT‐10 MV, respectively.^19^ Early screening of GD using a self‐administered scale is important because mental health problems are a risk factor for developing GD, common comorbidities, and consequences of GD.^9^, ^20^, ^21^

In the comparison of the three diagnostic groups (depression, ASD, and ADHD), the mean total scores on the GAMES Test and IGDT‐10 MV were significantly higher in the ADHD group than in the depression and ASD groups. The ADHD group had longer mean gaming times on weekdays and weekends. These results are consistent with previous studies showing an association between ADHD and GD.^9^, ^13^, ^20^, ^22^ Several studies have investigated the relationship between ADHD and GD. Almost all the participants consistently reported a link between the two conditions. Among the rates of IGDT‐10 OV positivity among the three groups, the ASD group had the lowest rate. However, since only eight cases were screened positive by IGDT‐10 OV and we divided them into three groups, it is difficult to draw any conclusions due to the small sample size.

School refusal was more frequent in the depression group than in other groups. This result indicates that, as is generally known, there is often comorbidity between depression and school refusal. When we performed two group comparisons between the presence and absence of school refusal in overall subjects, no significant differences were found for the GAMES Test (2.8 ± 2.3 vs 2.5 ± 2.2, *p* = 0.5911), IGDT‐10 MV (3.5 ± 2.0 vs 3.3 ± 2.4, *p* = 0.6846), and IGDT‐10 OV (1.2 ± 1.6 vs 0.9 ± 1.4, *p* = 0. 3281). The finding that the mean total scores on the GAMES Test and IGDT‐10 OV/MV were not higher in the depression group with the highest rate of school refusal than in the other groups also suggests that there was only a weak correlation between school refusal and GD. School refusal and subsequent severe social withdrawal (*hikikomori*) have long been common issues that are difficult to resolve in Japan and should be addressed by psychiatrists.^23^, ^24^, ^25^ As the Internet has become an integral part of daily life, it is often assumed to be the cause of school refusal and *hikikomori*.^26^ However, the results of the present study suggest that GD is unlikely to be the cause of those two conditions. The reason for this was considered after discussion among the co‐researchers of this study to be that, in many cases, adolescents were preoccupied with various online activities, such as online gaming. The study participants seemed addicted to the Internet rather than gaming online.

The IGDT‐10 was developed on the basis of the diagnostic criteria for IGD in the DSM‐5, whereas the GAMES Test was developed on the basis of the diagnostic guidelines for GD in the ICD‐11. Although IGD and GD are similar concepts, IGD is exclusively used for online gaming, and GD is applicable to offline gaming.^27^, ^28^ While there are numerous screening tools for GD,^15^ the GAMES Test, developed in Japan, has a unique feature in scoring: it assigns 1 or 2 points based on the amount of time spent on gaming, with 0 points for less than 2 h per day, 1 point for 2–6 h, and 2 points for more than 6 h. The relationship between the length of gaming time and the severity of GD remains controversial. However, a face‐to‐face diagnostic assessment of a large sample in Japan (*n* = 325) found that many individuals who played games for 6 or more hours on weekdays were diagnosed with GD based on the ICD‐11 criteria.^17^ In this study, the correlation between the GAMES Test and IGDT‐10 MV was stronger than that between the GAMES Test and IGDT‐10 OV. When we used self‐administered scales for early screening of GD in Japan, the GAMES Test or IGDT‐10 MV seemed to be better.

This study had several limitations. As this was a single‐site cross‐sectional study, our results cannot be generalized. The sample size was not large enough to obtain conclusive results. None of the participants participated in the face‐to‐face interviews. These results may have been affected by the COVID‐19 pandemic.

## CONCLUSION

Our results demonstrate that the positive screening rate for GD among adolescents with mental health issues was significantly higher than that observed in the general population. Since excessive gaming has a wide variety of adverse effects on youth mental and physical health, the early detection of probable GD is crucial. Therefore, GD screening should be performed in the daily clinical practice.

## AUTHOR CONTRIBUTIONS

Masaru Tateno, Takanobu Matsuzaki, and Ayumi Takano developed the conception and design of the study. Masaru Tateno, Takanobu Matsuzaki, and Susumu Higuchi obtained funding. Susumu Higuchi supervised the study. Masaru Tateno and Yukie Tateno collected the data. Masaru Tateno and Takahiro A. Kato contributed to the analysis and interpretation of data. Masaru Tateno drafted the article. All authors contributed to the revision and approved the final version of the article. All authors had full access to the data in this study, and take responsibility for the integrity of the data and the accuracy of the data analysis.

## CONFLICT OF INTEREST STATEMENT

The authors declare no conflicts of interest.

## ETHICS APPROVAL STATEMENT

The Ethics Committee of Tokiwa Hospital approved this study. Informed consent was obtained from the participants and their guardians. This study was conducted in accordance with the Declaration of Helsinki guidelines.

## PATIENT CONSENT STATEMENT

N/A.

## CLINICAL TRIAL REGISTRATION

N/A.

## Data Availability

The data that support the findings of this study are available from the corresponding author, Masaru Tateno, upon reasonable request. Requests will be considered on an individual basis based on ethical considerations and study requirements.
